# PBRM1 suppresses bladder cancer by cyclin B1 induced cell cycle arrest

**DOI:** 10.18632/oncotarget.3879

**Published:** 2015-04-19

**Authors:** Li Huang, Yang Peng, Guangzheng Zhong, Weibin Xie, Wen Dong, Bo Wang, Xu Chen, Peng Gu, Wang He, Shaoxu Wu, Tianxin Lin, Jian Huang

**Affiliations:** ^1^ Department of Urology, Sun Yat-Sen Memorial Hospital, Sun Yat-Sen University, Guangzhou 510120, China; ^2^ Guangdong Provincial Key Laboratory of Malignant Tumor Epigenetics and Gene Regulation, Sun Yat-Sen Memorial Hospital, Sun Yat-Sen University, Guangzhou 510120, China

**Keywords:** bladder cancer, PBRM1, cell cycle, cyclin B1

## Abstract

Growing evidence indicates that dys-regulation of PBRM1 contributes to tumorigenesis. However, little is known about the biological function of PBRM1 in the development or progression of bladder cancer. In this study, we aimed to elucidate the pathophysiological role of PBRM1 in bladder cancer. We assessed the expression of PBRM1 in 64 bladder cancer tissue samples with matching normal tissues. We explored the biological functions of PBRM1 both *in vitro* and *in vivo*. Mutational status of PBRM1 was analyzed. Effect of PBRM1 on cell cycle was evaluated. qRT-PCR and Western blot were carried out to evaluate the expression of cyclins affected by PBRM1. Our results showed that PBRM1 expression was significantly reduced in bladder cancer cells and tissues compared to their normal counterparts. The reduced expression of PBRM1 was associated with advanced tumor stage, low differentiation grade and worse patient outcome. Further functional analysis demonstrated that PBRM1 suppressed bladder cancer cell proliferation, migration, colony formation *in vitro* and tumorigenicity *in vivo*. Genetic alteration analysis showed no amino-acid sequence altering mutations. We found that PBRM1 could block the G2/M transition by repressing cyclin B1. Our data indicated that PBRM1 functions as a tumor suppressor in bladder cancer by repressing cyclin B1 expression.

## INTRODUCTION

Bladder cancer is one of the major causes of global cancer mortality, with an estimated 72,570 new cases and 15,210 deaths in 2013 alone [1]. There are two types of bladder cancer: non-muscle invasive tumor (70%) and muscle-invasive tumor (30%). Patients with non-muscle invasive bladder cancer frequent relapse after treatment and approximately 10-20% of them progress to muscle-invasive bladder cancer [2]. Although conventional clinical variables, such as the tumor stage, grade, tumor size, number of tumors are generally regarded as prognostic factors, biomarkers are needed in clinical practice [3]. Molecular analysis has identified some altered genes that have potential to be used as therapeutic targets in patients with bladder cancer, including HRAS, FGFR3, PIK3CA, HER2, p53 and RB [4-7]. However, there is still rooms in understanding the molecular mechanisms in bladder cancer and identification of novel functional molecular factors is of importance.

SWI/SNF (SWItch/Sucrose NonFermentable) complex has been involved in a variety of biological processes and represents a novel link between chromatin remodeling and tumor suppression owing to its recurrent mutations in a broad spectrum of cancer types [8-14]. Existing evidence indicated that SWI/SNF component has gene mutations in about 19% of human tumors, and acts as tumor suppressors [14], indicating that subunits of SWI/SNF complex are involved in human cancer pathogenesis. Mutation of the SWI/SNF complex may lead to a state called epigenomic instability, in which cells could be selected for growth advantage and malignant transformation [15]. Recently, the SWI/SNF complex subunit PBRM1 has been suggested to exert its tumor suppressive functions in renal cell carcinoma [10] and breast cancer [16]. PBRM1 maps to chromosome 3p21, a region where structural abnormalities were also frequently detected in bladder cancers [17], implying a strong potential tumor suppressor effect in bladder cancer. However, biological functions and prognostic values of PBRM1 in bladder cancer remain largely unexplored.

In this report, we found that PBRM1 was down-regulated in bladder cancer cell lines and tissues compared to normal cell line and normal tissue. In addition, low PBRM1 expression was associated with shorter overall survival in bladder cancer patients. PBRM1 suppressed bladder cancer cell growth *in vitro* and tumorigenicity *in vivo*. We also found that PBRM1 induced G2 cell arrest by repressing cyclin B1. Taken together, this report indicated that PBRM1 exerted a tumor suppressing role and induced cell cycle arrest in bladder cancer, which might partly be due to suppressing cyclin B1.

## RESULTS

### Expression of PBRM1 is lower in bladder cancer cells and reduced in bladder cancer tissues compared with their normal entities

**Figure 1:**
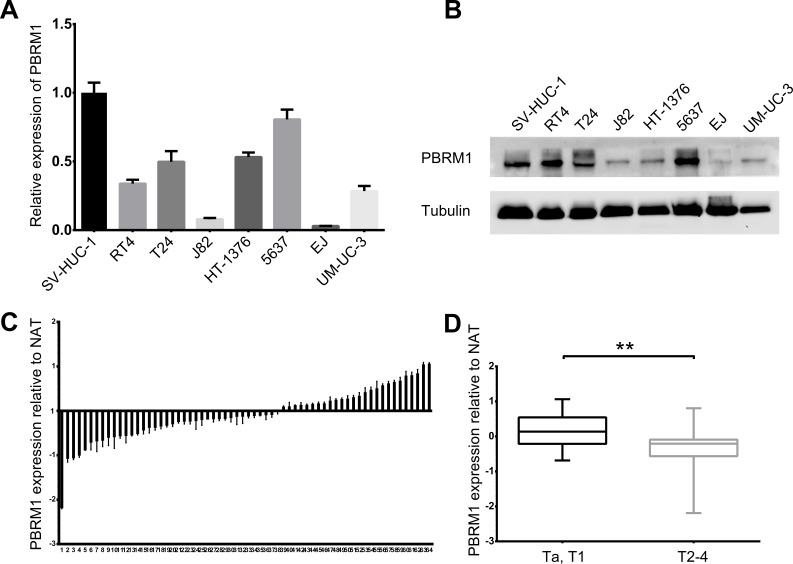
Reduced expression of PBRM1 in human bladder cancer cell lines and tissues **A.** Real-time PCR analysis of PBRM1 expression level in 7 bladder cancer cell lines (EJ, J82, UM-UC-3, RT4, T24, HT-1376, 5637) relative to normal uroepithelial cell line SV-HUC-1. **B.** Expression of PBRM1 in bladder cancer cell lines detected by Western blotting. Tubulin was used as internal reference for PBRM1 protein. **C.** Real-time PCR analysis in 64 cases of bladder cancers tissues relative to normal adjacent tissues (NAT), showing low PBRM1 expression in 38 (59.4%) cases (*p* < 0.05). Columns below the X-axis indicate low expression of PBRM1; those above the X-axis indicate over-expression of PBRM1. (D) PBRM1 expression in bladder cancers (Ta, T1 versus T2-4), showing decreased expression of PBRM1 in T2-4 versus Ta, T1. Data were presented as mean ± SD, from triplicate experiments. Significant differences are indicated by **p < 0.01.

We examined the expression of PBRM1 in both human bladder cancer cell lines and normal uroepithelial cell line SV-HUC-1 by qRT-PCR and Western blotting. We found that mRNA and protein expression of PBRM1 was lower in the bladder cancer cell lines compared with normal uroepithelial cell line (Figure 1A and 1B).

**Figure 2:**
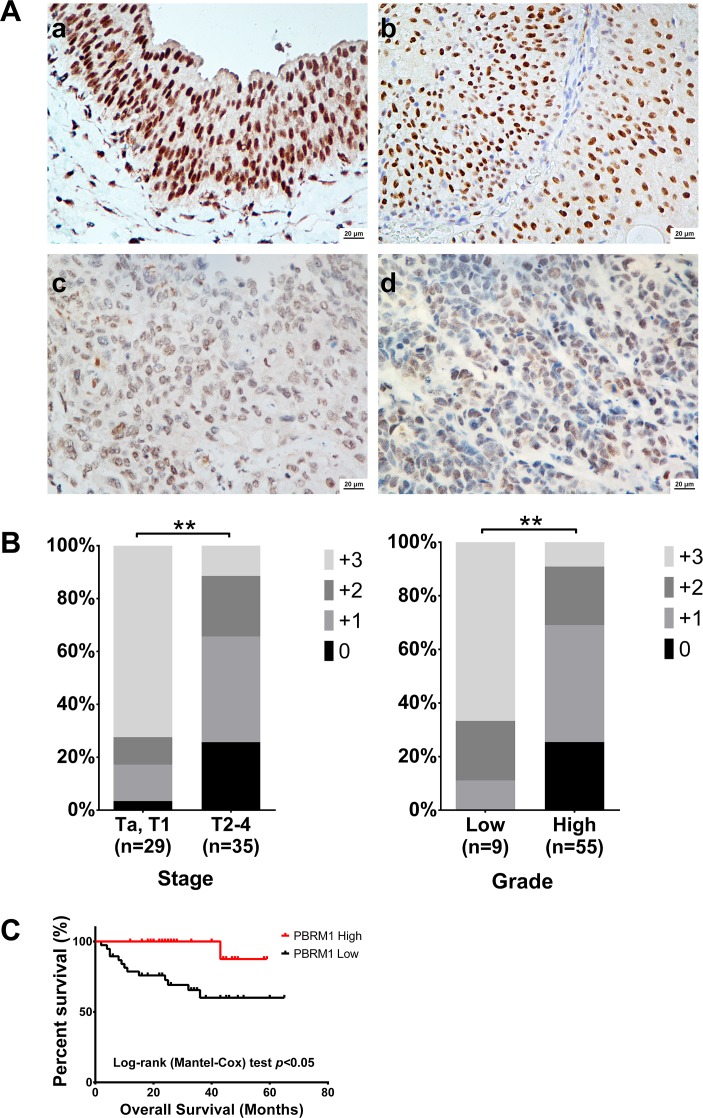
PBRM1 immunohistochemistry of bladder cancer tissue and its association with tumor stage, grade and overall survival **A.** PBRM1 expression in bladder cancer tissues detected by immunohistochemistry. a) normal bladder epithelium tissue, b) low grade bladder cancer tissue, c) and d) high grade bladder cancer tissue. **B.**Associations between PBRM1 expression patterns and tumor stage and grade. **C.** Kaplan-Meier curves for overall survival. Data were presented as mean ± SD from triplicate experiments. Significant differences are indicated by **p < 0.01.

Then we analyzed the mRNA and protein expression of PBRM1 in 64 paired bladder cancer tissues and adjacent non cancerous tissues by qRT-PCR and immunohistochemistry. Compared with their non cancerous counterparts, significantly lower expression of PBRM1 mRNA was observed in 59.4% (38/64) of bladder cancer samples (Figure 1C). In addition, PBRM1 mRNA expression was found to be reduced in muscle invasive bladder cancer compared with non-muscle invasive bladder cancer (Figure 1D). To further confirm the finding, immunohistochemistry using PBRM1 primary antibody was applied to examine the expression of PBRM1 in bladder cancer tissue as well as normal bladder uroepithelium. Results of PBRM1 immunohistochemical staining were depicted in Figure 2. Consistent with the qRT-PCR findings, PBRM1 had the highest expression in normal uroepithelium, and the expression decreased in superficial non-muscle-invasive and muscle-invasive bladder cancer tissues (Figure 2A).

These results suggested that reduced PBRM1 expression was a frequent event in human bladder cancer and might be involved in bladder carcinogenesis.

### Decreased PBRM1 expression is associated with poor differentiation, late tumor stage and worse patient outcome

To explore whether the differential expression of PBRM1 was associated with clinicopathological parameters, associations between mRNA and protein expression of PBRM1 and its clinical outcomes were investigated.

Statistical analysis revealed that PBRM1 mRNA expression was positively associated with tumor stage (Table 1). PBRM1 protein expression was positively associated with tumor stage and tumor grade (Figure 2B). Both correlations were of statistical significance. No statistical correlation with patients' gender and age was observed. As expected, there was also a significant association between PBRM1 expression and overall survival in patients with bladder cancer (Figure 2C). The univariate analysis revealed that PBRM1 expression correlated significantly with the overall survival of bladder cancer patients (*p* = 0.007). PBRM1 expression (HR = 0.093, 95%CI, 0.011-0.812, *p* = 0.032) and metastasis (HR = 44.729, 95% CI, 8.134-245.966, *p* < 0.001) were found to be independent predictors for overall survival (Table 2).

**Table 1:** Patients and pathological characteristics and the association between different variables and PBRM1 gene expression

Characteristic	Patient frequency (%)	PBRM1 expression		Chi-square	*p* value
		Low	High		
Total	64	38	26		
Gender					
Male	59 (922)	37	22	3.486	0.062
Female	5 (7.8)		4		
Age (yr)					
<65	38 (59.4)	20	IS	1.763	0.184
>65	26 (60.6)	18	8		
Tumor stage					
Ta, TI	29 (45.3)	1 I	18	10.11	0.002^*^
T2-T4	35 (54.7)	27	8		
Tumor grade					
G1	9 (14.1)	4	5	0.968	0.325
G2-G3	55 (85.9)	34	21		
Recurrence					
No	55 (85.9)	32	23	0.231	0.631
Yes	9(14.1)	6	3		
Metastasis					
No	50 (78.1)	27	23	2.738	0.098
Yes	14 (21.9)	11	3		
Lymph node status					
N0	47 (73A)	25	22	2.805	0.094
N1, N2	17 (26.6)	13	4		

Results were considered statistically significant at *p <0.05.

**Table 2:** Univariate and multivariate analysis for overall survival in bladder cancer

Variable	Univariate analysis	Multivariate analysis	
	*p* value	*p* value	HR (95%Cl)
Gender (male vs. female)	0.920		
Age (<65 years vs. >65)	0.044		
Tumor stage (Ta, T1 vs. T2-T4)	0.001		
Tumor grade (G1 vs. G2-G3)	0.491		
Recurrence (No vs. Yes)	0.000		
Metastasis (No vs. Yes)	0.000	0.000	44.729 (8.134-245.966)
Lymph node status (N0 vs. N1, N2)	0.000		
PBRM1 expression (Low vs High)	0.007	0.032	0.093(0.011-0.812)

The results above indicated that patients with high PBRM1 expression tumors had a better prognosis than patients with low PBRM1 expression tumors.

### PBRM1 suppresses cell proliferation, migration and colony formation *in vitro* and tumorigenicity *in vivo* with bladder cancer cells

**Figure 3:**
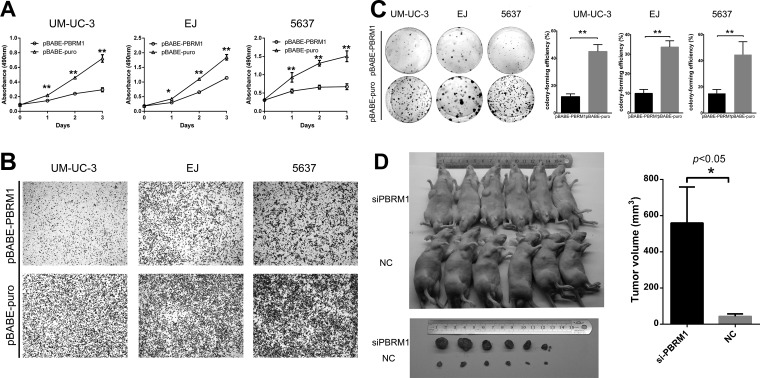
PBRM1 suppresses cell proliferation, migration and colony formation of bladder cancer cell *in vitro* and tumorigenicity *in vivo* **A.** High expression of PBRM1 inhibited cell growth and proliferation in bladder cancer cell UM-UC-3, EJ and 5637. Absorbance values were used to indicate cell numbers. **B.** Representative images of transwell assay after up-regulation in bladder cancer cell UM-UC-3, EJ and 5637. **C.** High expression of PBRM1 inhibited colony formation of bladder cancer cells. Colony forming efficiency was shown in the right column. Colony-forming efficiency was calculated as colonies/plated cells ×100%. **D.** Representative images of tumorigenicity assay performed in nude mice. Analysis of tumor volume in PBRM1 knockdown compared with control group was shown in the right column (*n* = 6). All results were presented as the means ± SD from 3 independent experiments. (*p < 0.05; **p < 0.01).

To examine the potential role of PBRM1 in tumorigenesis, we first evaluated the effect of PBRM1 on the growth and clonogenicity of cancer cells *in vitro*. We up-regulated PBRM1 expression by transfecting pBABE-PBRM1 or pBABE-puro in UMUC-3, EJ and 5637 bladder cancer cells. We confirmed the up-regulation of PBRM1 mRNA and protein in those cell lines (Supplement Figure 1A and B). We observed that up-regulation of PBRM1 mRNA and protein resulted a significant suppression in cell proliferation (Figure 3A), migration (Figure 3B) and colony formation (Figure 3C).

Abrogation of PBRM1 expression via siRNA was investigated to assess the possible consequences of PBRM1 silencing. Down-regulation of PBRM1 was confirmed (Supplement Figure 1C and D). As expected, down-regulation of PBRM1 mRNA and protein led to a significant enhancement in cell proliferation, migration and colony formation (Supplement Figure 2).

To study the effect of PBRM1 on the tumorigenicity of bladder cancer *in vivo*, PBRM1 siRNA and normal control (NC)- transfected UM-UC-3 cells were injected subcutaneously into the anterior flank of the nude mice. Compared with NC-transfected UM-UC-3 cells, si-PBRM1-transfected cells led to an increased size of tumor volume (*p* < 0.05) (Figure 3D).

Collectively, both *in vitro* and *in vivo* studies supported a growth inhibitory effect of PBRM1 on bladder cancer cells. These data suggested that PBRM1 had a tumor suppressor role in bladder cancer.

### Genetic alterations of PBRM1

**Figure 4:**
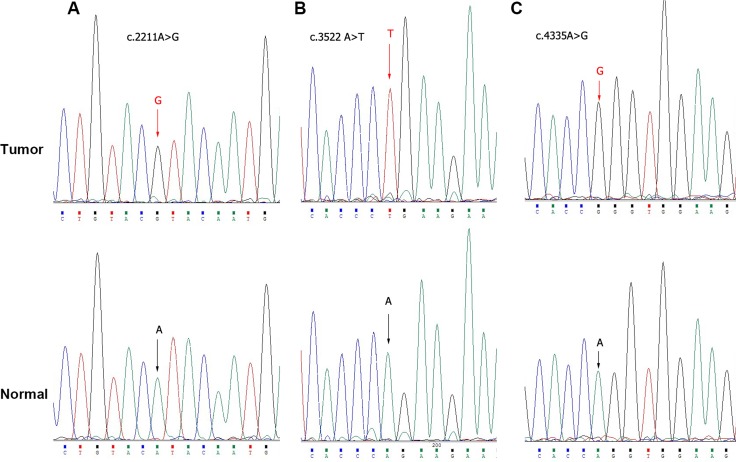
Exome sequencing of PBRM1 by Sanger sequence in bladder cancer tissues Sanger sequence analysis showed genetic alterations **A.** c.2211A>G, **B.** c.3522A>T and **C.** c.4335A>G respectively.

The above studies indicated that PBRM1 played a role in growth inhibition of bladder cancer. Recently, PBRM1 had been demonstrated to exert tumor suppressing properties owing to its frequent mutations in various cancer types, including renal cell carcinomas and breast cancer [10, 16]. These findings prompted us to study the mutation status of PBRM1 in bladder cancer. We amplified PBRM1 genome DNA by PCR and then sequenced it in 31 paired bladder cancer tissues. We found three SNPs (c.2211A>G (5/31), c.3522A>T (14/31), c.4335A>G (4/31)) (Figure 4, Supplement table 2), but no amino-acid sequence altering mutations.

This result indicated that no amino-acid altering mutations of PBRM1 could be detected in the bladder cancer tissues examined. This might suggest that mutation of PBRM1 was not a possible contributing pathogenesis of bladder cancer.

### Exogenous expression of PBRM1 induces cell growth arrest in G2 phase

**Figure 5:**
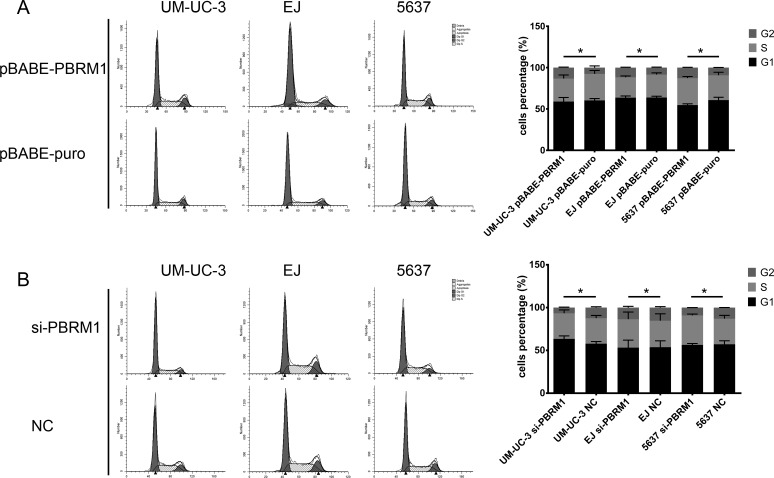
Flow cytometry analysis of cell cycle distribution after transfection and histograms of each phase in cell cycle of bladder cancer cells **A.** In UM-UC-3, EJ and 5637, up-regulation of PBRM1 increased cell proportion in G2 phrase. **B.** In UM-UC-3, EJ and 5637, down expression of PBRM1 decreased cell proportion in G2 phrase. All results were expressed as means ± SD, *n* = 3. (*p < 0.05).

Previous studies identified PBRM1 involved in pathways associated with cell cycle control [16, 18]. To explore the mechanisms underlying PBRM1 suppressed tumor growth, we investigate the impact of PBRM1 on cell cycle progression. We transfected pBABE-PBRM1 or pBABE-puro and si-PBRM1 or NC into UM-UC-3, EJ and 5637 separately. After transfection, cell cycle analysis was performed using flow cytometry. The results showed that UM-UC-3, EJ and 5637 cells with PBRM1 over expression have higher proportions of cells in G2 phase compared to control groups, while fewer cells in G2 phase were detected in siRNA groups. These results revealed that enforced expression of PBRM1 caused a marked accumulation of G2 population in different cell lines compared to that of the controls (Figure 5).

Taken together, these data indicated that PBRM1 played a role in regulating the G2/M transition of the cell cycle when introduced into bladder cancer cells.

### Cyclin B1 is suppressed by PBRM1 in bladder cancer cell lines and is required for G2 cell cycle arrest

**Figure 6:**
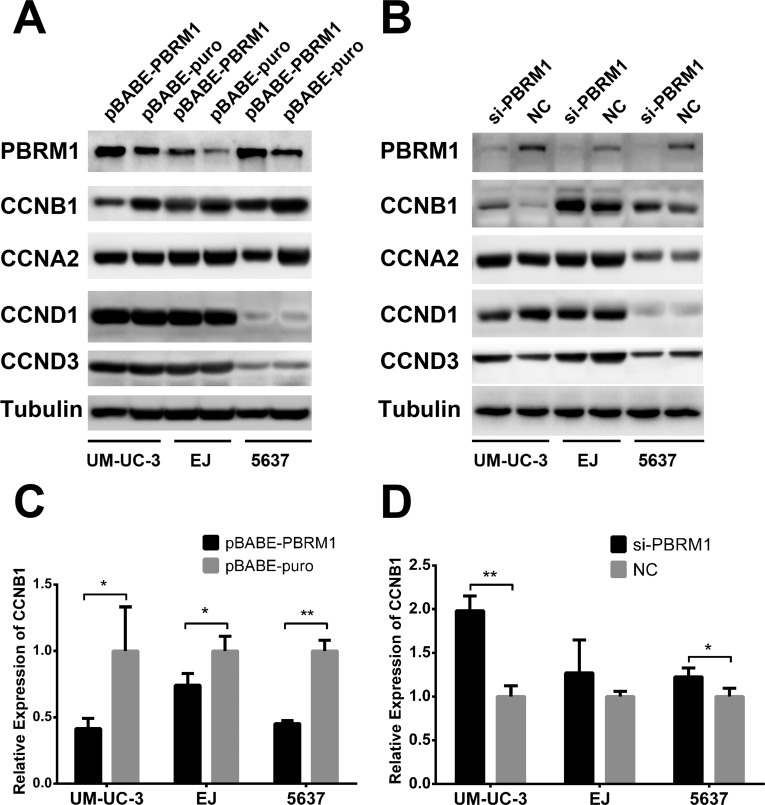
PBRM1 suppresses cyclin B1 expression in bladder cancer cell **A.** After up regulation of PBRM1, protein levels of cyclins (B1, A2, D1, D3) were assayed by Western blotting. **B.** After knockdown of PBRM1, protein levels of cyclins (B1, A2, D1, D3) were assayed by Western blotting. Tubulin was used as internal control. **C.**-**D.** PBRM1 inhibited cyclin B1 expression detected by qRT-PCR. (*p < 0.05; **p < 0.01).

To determine the signaling pathway through which PBRM1 mediates cell cycle regulation, we analyzed the protein levels of several cyclins (cyclin A2, D1, D3 and B1) in bladder cancer cells. We found that up-regulation of PBRM1 significantly decreased the protein level of cyclin B1 in UM-UC-3, EJ and 5637 cell lines (Figure 6A). On the contrary, knockdown of PBRM1 increased the expression of cyclin B1 protein (Figure 6B).

There were no significant changes in the protein level of other cyclins except cyclin B1, which was suppressed by PBRM1 for G2 cell cycle arrest.

To determine whether PBRM1 regulates cyclin B1 at the mRNA level, qRT-PCR was performed to measure mRNA levels of cyclin B1 in the presence or absence of PBRM1. We found that up-regulation of PBRM1 led to a reduction in the mRNA level of cyclin B1 and knockdown of PBRM1 led to an increased mRNA level of cyclin B1 (Figure 6C and 6D), suggesting that PBRM1 regulate the transcription of cyclin B1 at its promoter. The result of Western blotting analysis was corresponding to the result of qRT-PCR, showing decreased protein levels of cyclin B1 commensurate with the reduction in cyclin B1 mRNA expression. These results suggested that PBRM1 regulated cyclin B1 expression at the mRNA level.

## DISCUSSION

Our results demonstrated that reduced expression of PBRM1 was a central feature of bladder cancer. Univariate analysis indicated that reduced expression of PBRM1 was associated with tumor progression, emphasizing an important tumor suppressive role of PBRM1 in bladder cancer. PBRM1 was an independent prognostic indicator for overall survival. In a previous study, PBRM1 was not detected in 30.4% of clear cell renal cell carcinomas. PBRM1-negative expression was associated with high tumor stage, tumor recurrence, tumor-related death and indicating a poor prognosis in clear cell renal carcinoma [19]. These data supported our findings in bladder cancer and confirmed the possible role of PBRM1 as a useful tumor biomarker.

In this study, a series of functional studies were performed and showed that ectopic expression of PBRM1 suppressed bladder cell proliferation, cell migration and colony formation *in vitro* and tumorigenicity *in vivo*. Analysis of cell cycle distribution revealed that PBRM1 could increase cell proportion in G2 phase. PBRM1 significantly repressed the expression of cyclin B1 in bladder cancer cells. We suggested that reduced expression of PBRM1 disrupts cell cycle control, in turn promotes cell proliferation and facilitates the development of bladder cancer.

The mechanism of PBRM1 in tumor suppression is poorly understood despite extensive studies in recent years. PBRM1 mutation in various types of cancer indicated its involvement in carcinogenesis and its role as a tumor suppressor [10, 16]. These results indicated that PBRM1 suppresses tumor progression, which does not contradict to our present results in bladder cancer. Notably, PBRM1 is located at chromosome 3p21, a region where structural abnormalities were also detected in bladder cancers [17], implying a potential tumor-suppressive function of this gene. In our study, no amino-acid sequence altering mutations were detected in bladder cancer tissues. This result was in consistent with a previous study [20], suggesting that mutation of PBRM1 was unlikely to contribute to the pathogenesis of bladder cancer.

It is demonstrated that a defect in cell cycle control is an essential step during carcinogenesis. In human breast tumor, functional analyses revealed that truncated mutation of PBRM1 induced loss of cell cycle regulatory activity [16]. PBRM1 and another PBAF-specific subunit, BRD7, were identified important for prevention of faulty senescence by regulating p53 transcriptional activity, and when disrupted were found to promote proliferation [18]. Nevertheless, similar effects of PBRM1 in diverse cancer cells implicated its fundamental role in cell cycle regulation and tumorigenesis. In bladder cancer, cell cycle regulatory proteins, such as CABLES, Ki67, and cyclin D1, probably play a role in the tumorigenesis of bladder cancer [21]. We suggested that reduced expression of PBRM1 induces cell cycle arrest, in turn inhibits cell proliferation and suppresses the development of bladder cancer.

Cell cycle is controlled by cyclins and cyclin-dependent kinases [22]. Cyclin B1 is a key molecule for G2/M phase transition of the cell cycle and is needed for initiation of mitosis [23]. In this study, we showed that PBRM1 induces cell growth arrest in G2 phase. We found that cyclin B1 was suppressed by PBRM1 and was responsible for G2 cell cycle arrest.

An increasing body of data suggests that altered expression of cyclin B1 is a frequent event in tumor cells. Over expression of cyclin B1 has been demonstrated common in various tumor types, including colorectal, prostate, breast, esophagus, lung and head and neck cancers as well as Hodgkin and MALT lymphomas [24-34]. Cyclin B1 has been used as a prognostic indicator in those cancers. Cyclin B1 was considered as one of the genes strongly associated with disease recurrence. In bladder cancer, cyclin B1 was also found increased and the recurrence rate was significantly higher in cyclin B1-high patients than that of cyclin B1- low patients [35]. Because cyclin B1 has a direct effect on mitosis, overexpression of cyclin B1 causes uncontrolled cell growth and may promote malignant transformation. Cyclin B1 expression is regulated by p53 for G2/M transition [36] and cyclin B1 expression has also been detected in G1 phase [37]. This unscheduled expression may lead to substrate phosphorylation regardless of the cell-cycle phase and thus cause uncontrolled cell-cycle progression. This might be one of the mechanisms in genetic instability and carcinogenesis [38]. We also observed that cyclin B1 was suppressed by PBRM1, indicating its tumor promoting effect in bladder cancer.

In conclusion, we demonstrated for the first time that PBRM1 was present at low level in human bladder cancer tissues and cell lines. Our data implicated that PBRM1 might suppress cyclin B1 and exert its tumor suppressing role by inducing cell cycle arrest in bladder cancer. Based on these new findings, intervention with PBRM1 expression and function may have its potential application in cancer therapy. Future study will be focus on better understanding and characterizing PBRM1, particularly on exploring the expression of PBRM1 to predict the progression of superficial non-muscle invasive disease.

## MATERIALS AND METHODS

### Cell lines and cell culture

Human bladder cancer cell lines (T24, 5637, HT-1376, UM-UC-3, RT4, EJ, J82) and SV-HUC-1 were obtained from American Tissue Type Culture Collection (ATCC, Manassas, VA, USA). The cells were cultured in a humidified air atmosphere of 5% CO_2_ at 37°C, and all media were supplemented with 10% fetal bovine serum (Hyclone, Logan, UT, USA) and 1% penicillin/streptomycin (Gibco, Life Technologies, USA). T24 was cultured in McCoy's 5a medium; 5637 was cultured in RPMI 1640 medium; J82, UM-UC-3 and HT-1376 were cultured in Eagle's minimum essential medium (EMEM) (Gibco); SV-HUC-1 was cultured in F-12K Medium and RT4, EJ were cultured in Dulbecco's modified Eagle's medium (DMEM) (Gibco).

### Sample description and preparation

A total of 64 bladder cancer samples from newly diagnosed patients (mean age 62 years, age range 26-78 years) who underwent surgery from August 2007 to February 2012 were obtained with informed consent from the department of urology at Sun Yat-sen Memorial Hospital of Sun Yat-sen University. The primary tumor samples together with normal adjacent tissues and matched peripheral blood were obtained from these patients. The bladder cancer samples were chemotherapy-naive surgical resection specimens. All the specimens were snap frozen in liquid nitrogen upon collection and immediately stored at −80 °C until needed. All tissues were microscopically confirmed by two independent pathologists. A signed written consent from each subject was obtained before recruitment for the study according to the regulations of the institutional ethics review board.

### Cell transfection

Plasmid pBABE-PBRM1 [16] (#41078) and pBABE-puro (#1764) were transfected into the bladder cancer cells UM-UC-3, EJ, 5637 using X-tremeGENE HP DNA Transfection Reagent (Roche Applied Science, Mannheim, Germany) according to the manufacturer's protocol. siRNAs designed to PBRM1 (si-PBRM1) and negative control siRNA (NC) (Genepharma, Shanghai, China) were transfected into the bladder cancer cells UM-UC-3, EJ, 5637 using Lipofectamine® RNAiMAX (Life Technologies). Transfection efficiency was confirmed by using qRT-PCR and Western blot (Supplement Figure 1). The sequences used for si-PBRM1 were, sense: 5′-GGUCGUUUAUCAGCAAUUATT-3′, antisense: 5′-UAAUUGCUGAUAAACGACCTT-3′; and for negative control, sense: 5′-UUCUCCGAACGUGUCACGUTT-3′, antisense: 5′-ACGUGACACGUUCGGAGAATT-3′.

### RNA extraction and quantitative real-time reverse transcription PCR

Total RNA was extracted from the patients' bladder samples or cell lines using TRIzol reagent (Life technologies, Carlsbad, California, USA) according to the manufacturer's protocol. RNA electrophoresis was performed to inspect RNA integrity. Reverse transcription was performed using M-MLV reverse transcriptase (Life technologies) and quantitative real-time reverse transcription PCR (qRT-PCR) was performed to determine expression level of PBRM1 by using the SYBR green assay (Roche Applied Science, Mannheim, Germany) on a Roche LightCycler 480 machine (Roche Applied Science).

qRT-PCR was performed as followed: an initial pre-denaturation step for 30 seconds at 95°C, followed by amplification of 40 cycles at 95°C for 5 seconds and at 60°C for 20 seconds, melting curve analysis was performed at the end. All reactions were done in a 20 μl reaction volume in triplicate. The expression level of the PBRM1 was evaluated using the comparative Ct method. GAPDH was used as an internal control. The primers used for PBRM1 were: sense, 5′-AAGAAGAAAGAGCTTGCCAG-3′; antisense, 5′-TCTCGAGCTTCAAGAACAAC-3′; and primers for GAPDH the primers were: sense, 5′-GAAGGTGAAGGTCGGAGTC-3′, antisense, 5′-GAAGATGGTGATGGGATTTC-3′.

### Cell proliferation assay

After 24 hour transfection, bladder cancer cells were plated into 96-well plates at destiny of 1×10^3^ or 2×10^3^cells/well and incubated overnight. The cell viability was determined using the MTS assay (Promega, WI, USA) according to the manufacturer's protocol. OD value at the wavelength of 490nm was recorded through SpectraMax M5 reader (Molecular Devices. USA).

### Cell migration assay

After 24 hour transfection, bladder cancer cells were collected and suspended in 100 μl serum-free medium and then plated (1×10^4^ cells) in the upper compartment of Transwell plates (Corning, NY, USA). Transwell inserts were then placed into the lower compartment of a 24-well plate containing 600 μl of the medium with 20% FBS as the chemo-attractant. After a 24 hour incubation period, the cells remaining on the top surface of the membrane were removed and the cells on the lower surface were fixed in 100% methanol for 30 minutes, followed by staining with 0.1% crystal violet solution for 30 minutes. Cells that stained purple were defined as positive and the images were captured using a microscope (10×) (Olympus, Center Valley, PA, USA).

### Colony formation assay

Transfected bladder cancer cells were collected and placed into fresh six-well plates (1000 cells/well). The cells were cultured for 2 weeks to form colonies. Colonies were fixed with 100% methanol and stained with 0.1% crystal violet in 20% methanol for 15 minutes. Colony-forming efficiency was calculated as colonies/plated cells ×100%.

### Tumorigenicity in BALB/c-nude mice

All experimental procedures involving animals were in accordance with the Guide for the Care and Use of Laboratory Animals (NIH publication No. 80-23, revised 1996) and were performed according to the ethical guidelines for animal experiment of Sun Yat-sen University. All surgery was performed under sodium pentobarbital anesthesia, and efforts were made to minimize animal suffering. Male BALB/c-nude mice, aged 4-5 weeks were purchased from the Experimental Animal Center of Sun Yat-sen University. PBRM1 siRNA or NC transfected UM-UC-3 cells (5×10^6^) were suspended in 100μl PBS and then injected subcutaneously into the anterior flank of the nude mice. Tumor size was measured in perpendicular dimensions every three day. Animals were sacrificed on day 21 and tumor volume was calculated by the formula: Tumor volume (V) = 1/2 × (tumor length) × (tumor width)2.

### DNA extraction and PBRM1 mutation analysis

Total DNA was extracted from the patients' bladder cancer samples using the QIAamp reagent (QIAGEN, Germantown, MD, USA) according to the manufacturer's protocol.

Mutation analysis of the entire coding regions of PBRM1 was performed using polymerase chain reaction amplification and bidirectional Sanger sequencing. If mutations were successfully confirmed in the tumors, the same primer pairs were used to amplify the normal DNA from matched peripheral blood of the same subjects to determine the somatic statuses of the observed mutations. Primer sequences are provided in Supplement Table 1.

### Cell cycle analyses

After 48 hour transfection, a total number of 5×10^5^ cells were washed with PBS and fixed with frozen 70% ethanol in PBS at 4°C overnight. Then, fixed cells were treated with DNA-staining solution (3.4 mmol/L Tris-Cl (pH 7.4), propidium iodide (PI), 0.1% Triton X-100 buffer, and 100 mg/mL RNase A). Stained cells were subjected to fluorescence-activated cell sorting (FACS) flow-cytometry analysis of cells percentage in each phase of the cell cycle.

### Protein extraction and immunoblot analyses

Corresponding cell lysates was extracted from bladder cancer tissues and cell lines using RIPA buffer (Thermo Fisher Scientific Inc., Rockford, IL USA) containing protease inhibitors cocktail (Roche) according to the manufacturer's protocol. Accurate protein concentration was determinate by Pierce BCA Reagents (Thermo Fisher Scientific Inc.). Western blotting was carried out using the same antibody as for the immunohistochemistry analysis (dilution: 1:1,000). Additional primary antibodies such as rabbit anti-tubulin (dilution: 1:1,000) were used.

Briefly, 30 μg of protein lysates from each sample was separated by electrophoresis in 10% sodium dodecyl sulfate (SDS) polyacrylamide gel before being transferred to polyvinylidene fluoride (PVDF) membranes (Millipore, Billerica, MA, USA) for 2 hours. Membranes were blocked using 5% bovine serum albumin (BSA), and incubated in TBST (Tris buffered saline with 0.05% tween) containing rabbit polyclonal IgG2a anti-PBRM1 (1:1000, Sigma, molecular weight: 181 kDa) or anti-tubulin (1:1,000; cell signaling technology, molecular weight: 55 kDa) overnight at 4°C. The membranes were then incubated with peroxidase-conjugated goat anti-rabbit immunoglobulin (1:1000, Cell Signaling Technology) as secondary antibody and visualized using a commercial ECL kit (Pierce, Rockford, IL, USA).

### Immunohistochemistry and scoring system

Paraffin-embedded, formalin-fixed tissues were cut into 5 μm section, placed on a polylysine-coated slide, deparaffinized in xylene, rehydrated using graded ethanol, quenched for endogenous peroxidase activity in 0.3% hydrogen peroxide and processed for antigen retrieval by microwave heating in 10 mM citrate buffer (pH 6.0). The sections were then incubated at 4°C overnight with PBRM1 rabbit polyclonal antibody (1:200, Sigma, Cambridge, MA, USA). Immunostaining was performed using the ChemMate TM DAKO EnVision TM Detection Kit (DakoCytomation, Glostrup, Denmark), which resulted in a brown precipitate at the antigen site. Subsequently, sections were counterstained with hematoxylin (Zymed Laboratories, South San Francisco, CA, USA) and mounted in nonaqueous mounting medium. The primary antibody was omitted for the negative controls.

PBRM1 staining was assessed on the basis of staining frequency (% positive area) and staining intensity. PBRM1 staining was scored as follows: +3, strongly positive staining in 51% to 100% of the cells; +2, moderately positive staining, 25% to 50% of the cells; +1, weakly positive staining, 10% to 24% of the cells; 0, negative staining. Only nuclear staining was scored. Normal bladder epithelial cells served as positive internal controls.

### Statistics

Statistical analyses were performed using SPSS 17.0 software (SPSS Inc.). All quantitative data were expressed as the mean ± standard deviation (SD) from at least three independent experiments. The differences between groups were analyzed using Student's *t* test when only two groups were compared. Contingency table analysis and Pearson Chi-square tests were used to analyze the associations between PBRM1 expression patterns and pathological parameters. Overall survival was estimated using the Kaplan-Meier method and analyzed for statistical differences using a log-rank test. A Cox proportional-hazard model was used for the multivariate analyses.

All statistical tests were two-sided. Differences were considered statistically significant at *p* < 0.05.

## SUPPLEMENTARY MATERIAL FIGURES AND TABLES


