# Supplementary material for: PBRM1 suppresses bladder cancer by cyclin B1 induced cell cycle arrest
Source: Oncotarget. 2015 Apr 19;6(18):16366–78. doi: 10.18632/oncotarget.3879 (PMC4599275; doi:10.18632/oncotarget.3879)
Supplement: Supplementary file 1 [file oncotarget-06-16366-s001.pdf]

PBRM1 suppresses bladder cancer by cyclin B1 induced cell cycle arrest

Supplementary Material

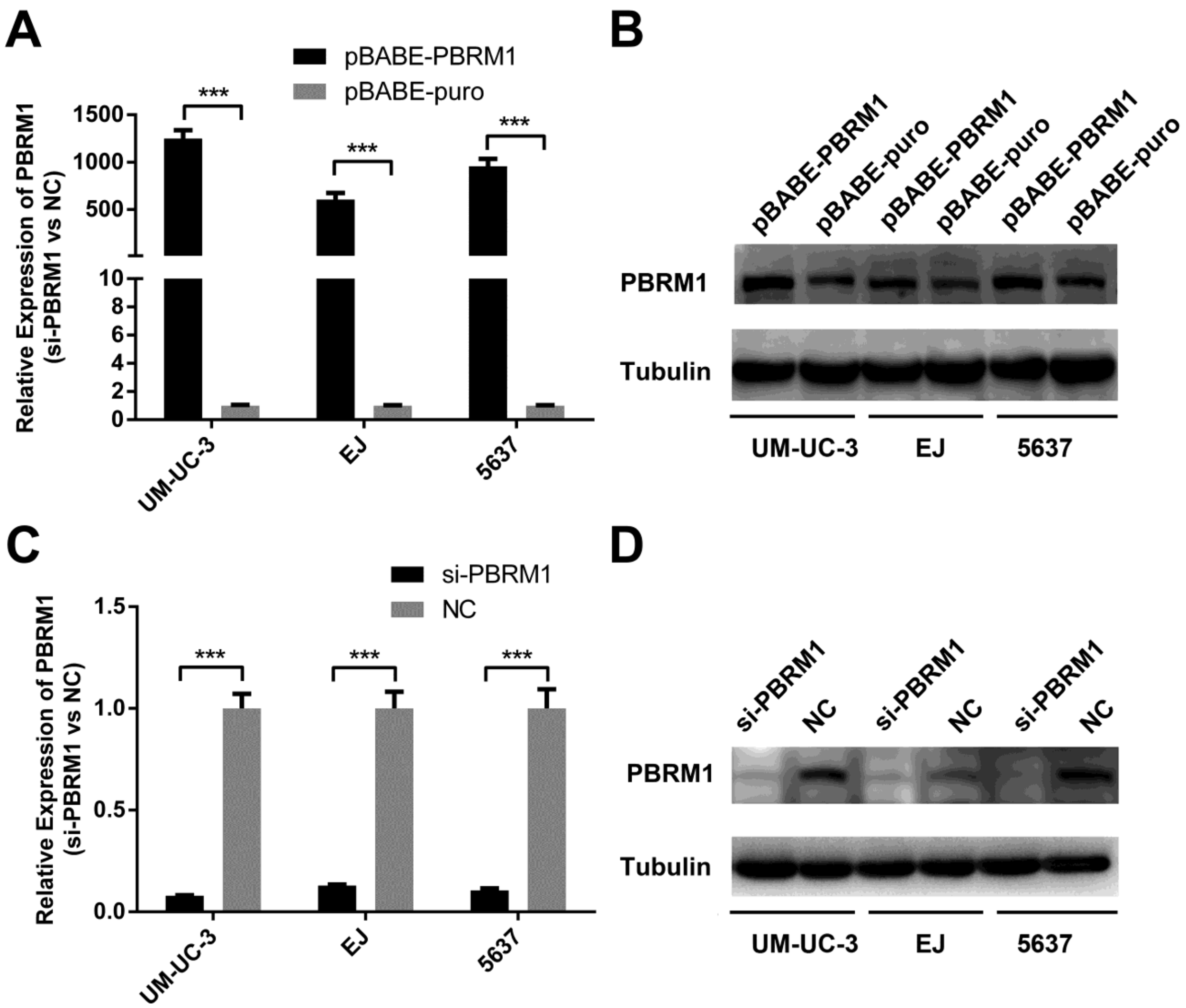

Supplement Fig 1: Validation of PBRM1 expression after transfection by qRT-PCR and Western blotting. (A, B) After transfection with pBABE-PBRM1, PBRM1 up-regulated expression level was confirmed by qRT-PCR (A) and Western blotting (B). (C-D) After transfection with siRNA, PBRM1 knockdown was confirmed by qRT-PCR (C) and Western blotting (D). Data were presented as mean ± SD from triplicate experiments. Significant differences are indicated by *** p<0.001.

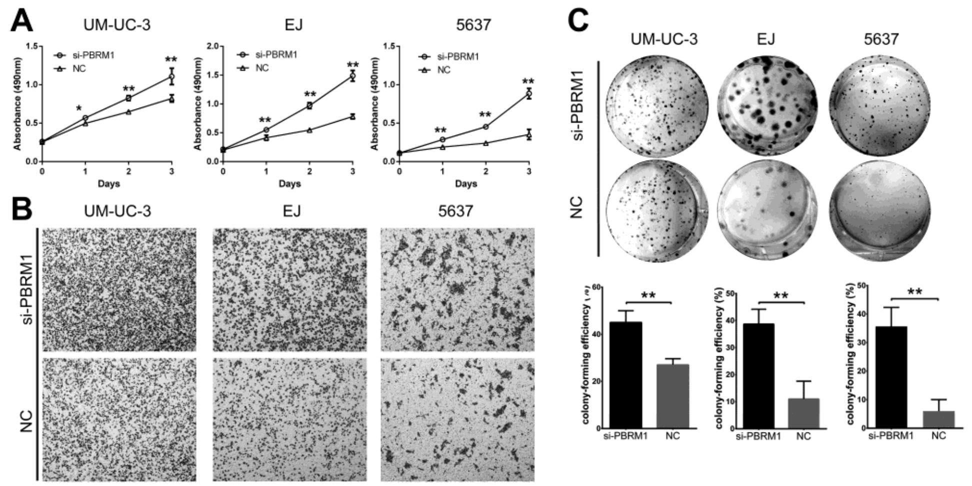

Supplement Fig 2: PBRM1 knockdown facilitated cell proliferation, migration and colony formation of bladder cancer cell *in vitro*. (A) Knockdown of PBRM1 promoted cell growth and proliferation in bladder cancer cell UM-UC-3, EJ and 5637. Absorbance values were used to indicate cell numbers. (B) Representative images of transwell assay after PBRM1 knockdown in bladder cancer cell UM-UC-3, EJ and 5637. (C) Knockdown of PBRM1 promoted colony formation in bladder cancer cells, and colony forming efficiency was shown in the right column. Colony-forming efficiency was calculated as colonies/plated cells ×100%. All results were presented as the means ± SD, resulting from triplicate performances. (*, $p<0.05$; **, $p<0.01$)

Supplement Table 1: PCR and sequence primers

No.	Forward	Reverse
1	AAACAAGGAAGTCCAGGGC	AAAAAGTGGAGATGCCTTGC
2	TTGGAAGCGGGATTTGGA	GGCACACGTTGTCCAGGAT
3	TTTGTCTGCAGGTTATATTTCACT	GTTTCAAGCAGGACTTTGTGTAG
4	CCCTCTAGATCTGAGTTGCCTG	ATCCTTCTTGCTCGTTCCAA
5	CCCAAATGTGACTTTGCTGA	AAGAGATTTTCAATTTTGTCTTCCTC
6	AAGTATCTTTTCATGTGTTTAATGGG	AAAAAGCACAAATACCTACCGA
7	CCATATGGACAACAGGTGAGC	AAACATGCAAAGAAACTCCAAAC
8	GAAATGTGCCTGGAAATATTCTG	TTGAAATAGCTTATTAAAAGTGTCCG
9	AAGTAAGCTTCAAAGTCCATGAAA	TAAAAATCATATGAATGTCCAGTCTC
10	GTTGCTGTTTTGAATTAGCTCTACA	CAACATCTTCCTTTTGAACTTACTTT
11	ATGGTTCTGATATAATAAATGTGCTG	TATAATCAGAAATGTCGGTAACCA
12	TACCTTAATGTAATGGTGCTTTTGC	TAATATTACTGCTGAGGGTGGGG
13	TTTTGTCATGCAGGCTTTTG	ATGTAGTAGTCATTTTCATCTGGGTC
14	CATGCAGTACTAAGGGTGCTTTATT	CTCTGCCATGTGTGCTGTG
15	TGACTTTACATGTTGTTCATATGTGT	AACTAACCTTGAATACTTGAGAGCC
16	GATTGATGGTGTATTTCCTAATTTTG	CAGAGTTCCTAATTTTGTAAACATCG
17	AAACTCTTTCCATGCTGCCT	TCATGGCACTGACAAAATCTG
18	GTTCAGCTTTTGTTTGGTTGG	TTCCTGTTTGGCTAAGGTTTTG
19	ACTCAGTTGTTTGAAAGGAGACA	AAAAAGCTTCACTACAGCTTGATTA

20	TGAAGATAGATATTTTGGAAGCTTGT	AGACATTTTCTTAAACCTACCTCATTC
21	AGTTGGGGGCATTAAGCTGT	CAGCAAATATAAAGGCATTAAGGG
22	TGCTTATAACTTCCAGCATGGTT	CTATAAGTACCCCTCTCCCGC
23	GGTTAAACCATCCAAAAAGGA	GGAACGTTTATCTTTATAATGTACTGC
24	CAAACTTCGGAAAGATACTCTTCA	TTCCATCTCATTGCGTCTACTC
25	GCATGTTGCAAATGGAATTAAA	TGTGACACTTGCCCAATAGGT
26	GTGTTCCTGGCTTCTGAAAAA	TCACAGCCCTCATCTCACTG
27	TTAAGTACAGAGATAAACTAAGGAGGC	GTTTTAAACCAGGATCTGCTAAGT
28	CAGGACTTTTGTTAAAACCTGC	CAGAAAAATCAGCAATCTCTTCTT
29	TTGGAATGTAGGTTTATAATGATGC	CTGTCCAGTTGGAACTGCTG
30	CCACAAAGATTCAAGGGCAG	TAATGCTGTTGGACTCCGC
31	GTACCAGGTCCTCTGGTCACT	AATAAACCATTTGACAACAGATTCTC
32	TTGAGCAGTTAACCAAATGTAATGT	TTTTGGAGAAATTTGCAGGG
33	GTAACTATGACCACAGTTTACTCCTTT	TGACAGGATATAAACCAGAACCA
34	AAGAAAAGTCTGCTTTCGTTTCTTAC	GTGTCACTTCAAATTTATGCTAAAGG
35	ATATTTAGTTCAATCTGGAAGGTTGT	GGTAATGAACAGTTAAAATTTGAGG

Supplement Table 2: PBRM1 mutations in bladder cancer

No.	Gender	Age	Stage	Grade	cDNA Annotation (NM_018313.4)	Protein Annotation (NP_060783.3)
1	Male	45	T1N0M0	High	c.3522A>T	p.Pro1174=
2	Male	62	T2aN0M0	High	c.3522A>T	p.Pro1174=
3	Male	54	T2bN0M0	High	−	
4	Male	71	T1N0M0	High	c.3522A>T	p.Pro1174=
5	Male	74	T2aN2M0	High	−	
6	Male	78	T3aN0M0	High	c.3522A>T; c.4335A>G	p.Pro1174=; p.Pro1445=
7	Male	69	T2bN0M0	Low	c.2211A>G	p.Thr737=
8	Male	60	T1N0M0	Low	c.3522A>T	p.Pro1174=
9	Male	73	T3bN2M0	High	−	
10	Female	53	T2aN0M0	High	−	
11	Male	68	T1N0M0	High	−	
12	Male	63	T2aN0M0	High	c.3522A>T; c.4335A>G	p.Pro1174=; p.Pro1445=
13	Male	61	T1N0M0	High	c.2211A>G	p.Thr737=
14	Male	77	T3bN2M0	High	c.3522A>T	p.Pro1174=
15	Male	49	T1N0M0	High	−	
16	Female	49	T1N0M0	Low	c.3522A>T	p.Pro1174=
17	Male	75	T3bN0M0	High	c.3522A>T; c.4335A>G	p.Pro1174=; p.Pro1445=
18	Male	53	T1N2M1	High	c.2211A>G	p.Thr737=
19	Male	35	T3aN1M0	High	c.3522A>T	p.Pro1174=
20	Male	47	T1N0M0	High	c.3522A>T	p.Pro1174=
21	Female	79	T3bN0M0	High	−	
22	Male	70	T2bN0M0	High	c.3522A>T	p.Pro1174=
23	Male	63	T3bN0M0	High	c.2211A>G; c.3522A>T	p.Thr737=; p.Pro1174=
24	Male	60	T3aN2M0	High	c.3522A>T; c.4335A>G	p.Pro1174=; p.Pro1445=
25	Male	66	T4aN1M0	High	c.2211A>G	p.Thr737=
26	Male	72	T3aN2M0	High	−	
27	Male	72	T4aN2M0	High	−	
28	Male	79	T2bN1M0	High	−	
29	Male	52	T3bN0M0	High	−	
30	Male	57	T1N0M0	Low	−	
31	Male	60	T3aN0M0	High	−	

−, no mutation was identifid in this patient; c., cDNA sequence; p., protein sequence.
